# *Listeria monocytogenes* Colonizes *Pseudomonas fluorescens* Biofilms and Induces Matrix Over-Production

**DOI:** 10.3389/fmicb.2018.01706

**Published:** 2018-07-31

**Authors:** Carmen H. Puga, Elias Dahdouh, Carmen SanJose, Belen Orgaz

**Affiliations:** ^1^Department of Food Science and Technology, Faculty of Veterinary, University Complutense of Madrid, Madrid, Spain; ^2^Department of Animal Health, Faculty of Veterinary, University Complutense of Madrid, Madrid, Spain

**Keywords:** *Listeria monocytogenes*, biofilms, food industry, *Pseudomonas*, persistence, CLSM

## Abstract

In food facilities, biofilms or their debris might act as *helpers* for attracting free floating microorganisms. In this sense, *Pseudomonas fluorescens*, a dense biofilm producer frequently isolated from food contact surfaces, could be a good candidate for sheltering other microorganisms, such as *Listeria monocytogenes*. The main objective of this work was to evaluate the ability of *L. monocytogenes* to colonize pre-established *Pseudomonas* biofilms. For this, the movement throughout mature *Pseudomonas* biofilms of a green fluorescent protein (GFP) – tagged strain of *L. monocytogenes* was tracked for 24 h by confocal laser scanning microscopy (CLSM). Moreover, in order to check the effect of the incorporation of *Listeria* on the overall matrix production, attached populations of both microorganisms and total biomass (cells + matrix) of the resulting biofilms were measured over time. Planktonic cells of *L. monocytogenes* efficiently migrated to preformed *P. fluorescens* biofilms. Moreover, they moved preferentially toward the bottom layers of these structures, suggesting some kind of tropism. When preformed *P. fluorescens* biofilms were conditioning the surfaces, the *L. monocytogenes* attached population was on average, 1–2 Log higher than when this organism grew on bare coupons. Furthermore, the arrival of *L. monocytogenes* to the already established *P. fluorescens* biofilms led to a matrix over-production. Indeed, biomass values [optical density (OD_595__nm_)] of the resulting biofilms were double those of the ordinary *L. monocytogenes–P. fluorescens* mixed biofilms (1.40 vs. 0.6). The fact that *L. monocytogenes* cells accumulate in the bottom layers of preformed biofilms provides this microorganism an extra protection toward physical–chemical damages. This might partly explain why this microorganism can persist in food industry environments.

## Introduction

*Listeria monocytogenes* presence in food processing facilities is a concerning issue for several reasons. Once established in a food processing plant, it can persist there for extended periods that could range from months to years, presumably hosted in and preserved by biofilms (Orgaz et al., 2013; Valderrama and Cutter, 2013). When transferred from contaminated surfaces to food, it might cause listeriosis, a relatively infrequent yet serious human disease, with high morbidity, hospitalization times, and mortality rates among vulnerable individuals (EFSA, 2017). Although many efforts are being done in order to control *Listeria*’s presence in food facilities, the truth is that in the last 5 years (from 2012 to 2016), there has been an increasing trend of confirmed listeriosis cases in the EU/EEA (EFSA, 2017).

*Listeria* is known to produce thin biofilms by itself, although very different structures have been described depending on the growing conditions (Rieu et al., 2008; Cherifi et al., 2017; Kocot and Olszewska, 2017). However, in the food industry, as in real environments in general, multispecies biofilms are prevalent (Elias and Banin, 2012; Burmølle et al., 2014; Jahid and Ha, 2014; Giaouris et al., 2015; Sanchez-Vizuete et al., 2015). In many studies investigating the presence of *L. monocytogenes* on food contact surfaces, the accompanying microbiota is often disregarded (Ortiz et al., 2010). The persistence of *L. monocytogenes* in food processing plants has been associated with several factors, including its ability to survive under harsh conditions (Carpentier and Cerf, 2011; Orgaz et al., 2013; Ferreira et al., 2014; Puga et al., 2016b; Rychli et al., 2016). However, it is likely that *L. monocytogenes* has partners or even *helpers* among the in-house microbiota that contribute to its persistence in certain niches. From an ecological point of view, *L. monocytogenes* can be considered *a cheater*, i.e., an organism that does not produce certain goods, but benefits from those produced by others (Cordero et al., 2012; Drescher et al., 2014). Among these goods, the extracellular matrix (ECM) is perhaps the most important. The ECM is the major component of the biofilms and is partly responsible for its resistance to different treatments (Flemming et al., 2016). In this context, the large amount of matrix produced by certain microorganisms can be regarded as a competitive advantage. That is the case when *Listeria* forms mixed biofilms along with species that produce copious amounts of ECM or extracellular polymeric substances (EPSs), such as *Pseudomonas* spp. (Huis in’t Veld, 1996; Liao, 2006). There, *L. monocytogenes*, a poor matrix producer, could find shelter inside the matrix produced by these organisms. *Pseudomonas* spp. are the most important spoilage microorganisms in many refrigerated products, in which they become the dominant species (Gram, 1993). Several species of this genus have been extensively isolated from dairy, fish, vegetable, and meat processing plants, with *P. putida* and *P. fluorescens* being the most prevalent (Chmielewski and Frank, 2003; Dogan and Boor, 2003; Caldera et al., 2016; Langsrud et al., 2016). Moreover, some studies regarding food plant-associated microbiota have frequently co-isolated *Pseudomonas* spp. and *L. monocytogenes* from the same food contact surfaces (Rodríguez-López et al., 2015; Langsrud et al., 2016).

A previous study of Puga et al. (2014) described that *L. monocytogenes* tends to get located in the deepest layers of the mixed biofilms when co-cultivated with *P. fluorescens*. These positions inside a biofilm, are more restrictive in terms of oxygen concentration and nutrients availability (Stewart and Franklin, 2008) but are tolerable for the facultative anaerobic *Listeria*. Besides, cells there, though constrained, are less exposed to biofilm damage (Sanchez-Vizuete et al., 2015; Flemming et al., 2016; Puga et al., 2016b).

Most of the studies on the development of multispecies biofilms rely on co-cultivation of different microorganisms in liquid media, but this situation may be not so common in real scenarios. For instance, surfaces conditioned by preformed biofilms that remain unremoved or just partially damaged after defective cleaning, might serve as anchorage points for free floating microorganisms (Castonguay et al., 2006; Klayman et al., 2009). In this case, it is unclear whether the outcome in terms of species distribution inside the biofilm would be the same as in the case of a co-cultivation. Would these new *dwellers* of the pre-established biofilms remain attached to the upper layers or would they penetrate into the matrix? Would the new *comers* have an effect, on the matrix production? Answering these questions is useful for the understanding of realistic biofilms, which need to be developed as targets for the improvement of already existing anti-biofilm strategies and for the design of new ones.

In this context, the main objective of this work was to evaluate the effect of a pre-established *Pseudomonas* biofilm on both the incorporation and positioning of *L. monocytogenes* in these structures. Confocal laser scanning microscopy (CLSM) was used to follow for 24 h the incorporation of a green fluorescent protein (GFP)-tagged strain of *L. monocytogenes* into the preformed, DAPI stained, thus blue *P. fluorescens* biofilms. Moreover, overall attached populations of *L. monocytogenes* and *P. fluorescens* and total biomass (cells + matrix) of the resulting biofilms were measured over a 96-h incubation period.

## Materials and Methods

### Bacterial Strains

*Pseudomonas fluorescens* ATCC 948^TM^ (isolated from dairy industry waste) and reference strain *L. monocytogenes* Scott A (serotype 4b, lineage I) were used as biofilm forming microorganisms. They were stored at -20°C in tryptone soya broth (TSB) (Oxoid) with 15% glycerol. Pre-inoculated cultures were incubated overnight while shaking (80 rpm) at 20°C in TSB to attain mid exponential phase. Cells were then harvested by centrifugation at 4000 *g* for 10 min, washed twice with sterile TSB and their suspension OD_600_ adjusted to 0.12. The two organisms were inoculated at an initial concentration of 10^4^ CFU mL^-1^, in both monospecies and dual-species cultures.

### Experimental System

**FIGURE 1:**
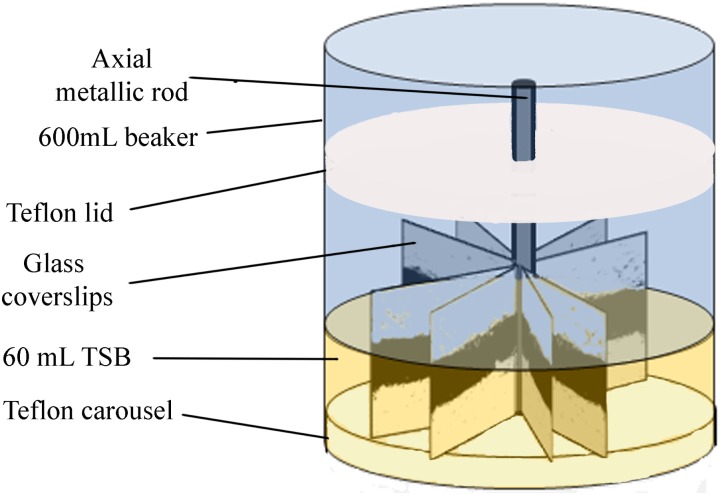
Experimental system. Carousel for biofilm development. Darker areas on the coverslips correspond to the more cell dense biofilm zones, near the air/water interface of the culture system. Reprinted from Puga et al. (2016b), with permission from Elsevier.

Biofilms were developed on commercial 22 mm × 22 mm, thin microscope borosilicate glass coverslips, as described by Orgaz et al. (2011). These coverslips provide single use, cheap, clean, and undamaged smooth surfaces, without scratches or other microtopographic irregularities. They are moderately more hydrophilic than stainless steel, but allow for more reproducible biofilms than reusable metal coupons. Sixteen coverslips held vertically by marginal insertion into the narrow radial slits of a Teflon carousel platform (6.6 cm diameter). The platform and its lid were assembled by an axial metallic rod for handling and placed into a 600-mL beaker (**Figure 1**). The whole system, i.e., coverslips, carousel, and the covered 600 mL beaker, were heat-sterilized as a unit before aseptically introducing 60 mL of inoculated TSB. For *L. monocytogenes–P. fluorescens* binary biofilms, both bacterial species were inoculated at the same time with the same initial concentration of 10^4^ CFU mL^-1^. Incubation was carried out at 20°C for 96 h in a rotating shaker at 80 rpm. Under these conditions, biofilm growth covered approximately 70% of the coverslip’s surface.

### Surface Conditioning

In order to evaluate the effect of pre-established biofilms on *L. monocytogenes* incorporation, those formed by *P. fluorescens* were used as substratum instead of the clean, bare borosilicate glass coverslips. Thus, using the experimental system described above, *warm* (20°C/48 h) and *cold* (4°C/10 days) *P. fluorescens* biofilms were previously developed. The whole carousels bearing them were washed twice in NaCl (0.9%) before being placed into a new beaker containing 60 mL of a *L. monocytogenes* suspension in TSB (at 10^4^ CFU mL^-1^). The system was incubated at 20°C for 96 h, under continuous shaking (80 rpm).

### Cell Recovery and Counting

For viable cell retrieval and count, attached cells were removed from the surfaces by swabbing both sides of the coverslips. Cells transferred into test tubes with 1.5 mL of peptone water, were vigorously mixed in a vortex stirrer to break up cell aggregates, decimally diluted in peptone water, and pour-plated. *P. fluorescens* and *L. monocytogenes* counts were quantified in selective media (*Pseudomonas* selective agar and PALCAM, respectively, Oxoid) wherein counting was performed after 48 h incubation at 30 and 37°C, respectively. For purity control, plating on Tryptone Soya Agar (TSA, Oxoid) was used to visually spot different colonies. For each type of biofilm, three independent experiments were carried out and two coverslips were taken from each carousel. Data thus correspond to an average of six samples.

### Biomass Determination

For biomass (cells plus EPS matrix) quantification, six coverslips of each type of biofilm were first dried and then stained for 2 min with a 1‰ Coomassie Blue (Brilliant Blue R, SIGMA) solution in an acetic acid/methanol/water (1:2.5:6.5) mixture. This step was repeated twice. Afterward, the stained coupons were immersed into 4 mL of the same solvent mixture and the biomass was detached with sterile cell scrapers. After full homogenization of this suspension, optical density (OD) was measured in a spectrophotometer using a wavelength of 595 nm. Bare coupons were stained and used as controls.

### Expression of Green Fluorescent Protein (GFP) in *Listeria monocytogenes* Scott A

Electrocompetent *L. monocytogenes* cells were prepared as previously described by Monk et al. (2008). The pLSI ROM–GFP plasmid used in this work contains the genes for GFP and resistance to Erythromycin (Fernández de Palencia et al., 2000). Fifty microliter of electro-competent cells were mixed with 2 μL of the plasmid preparation and transferred to a 0.2-cm electroporation cuvette. For electroporation, the electroporation system (Gene Pulser, BioRad) was used with the following settings: Resistance = 400 Ω, Capacitance = 25 μF, and Voltage = 2.5 kV. The average electroporation time was 4.5 s. The electroporation product was then immediately transferred into sterile BHI broth supplemented with 0.5 M sucrose and incubated at 37°C with gentle shaking for 1 h. The suspension was then centrifuged at 8000 *g* for 2 min. The supernatant was discarded and the pellet was spread on BHI agar containing 5 μg/mL Erythromycin and incubated at 37°C for 48 h. Colonies that grew after 48 h were suspended in sterile BHI broth and visualized under a fluorescent microscope in order to confirm the presence of GFP.

### Confocal Laser Scanning Microscopy (CLSM)

Preformed *warm* (20°C/48 h) and *cold* (4°C/10 days) *P. fluorescens* biofilms were used as adhesion substrates for the GFP-tagged *L. monocytogenes* Scott A strain. Interplay of both species was evaluated by time-series CLSM imaging using a FLUOVIEW^®^ FV 1200 laser scanning microscope (Olympus). Preformed *P. fluorescens* biofilms were first developed on 27 mm glass bottom culture dishes (cellview) (Nunc^TM^ Glass Bottom Dishes, 150686, Thermo Fisher Scientific), vertically held on the carousel platforms, as explained before for coverslips. Preformed *P. fluorescens* biofilms were rinsed with sterile 0.9% NaCl and stained with DAPI (D9542, Life Technologies), a cell permeable fluorescent probe that binds to DNA. Ten milliliter of 10^9^ CFU mL^-1^ GFP-tagged *L. monocytogenes* suspension were added to the glass bottom culture dishes with *Pseudomonas* biofilms for tracking *Listeria* movement. Thus, for image analysis, green corresponds to *Listeria* cells and blue corresponds to *Pseudomonas*. Most *Pseudomonas* spp. produce pyoverdin. This fluorescent siderophore is nevertheless produced under iron-deprived conditions (Trapet et al., 2016), which is not the case in our work, in which a rich medium was used for cultivation. Moreover, our parameters for detecting GFP fluorescence were λ_excitation_ = 488 nm and λ_emission_ = 520 nm. Pyoverdin fluorescence spectrum shows a maximum Excitation wavelength at 405 nm and a maximum Emission wavelength at 460 nm (Martin et al., 2011). Under these conditions, no residual fluorescence was observed in *P. fluorescens* biofilms.

Considering zero time the moment at which *Listeria* suspension was added to the cellview, *z*-stacks of a representative 0.12 mm × 0.12 mm region of the air–liquid interphase of the biofilm (**Figure 1**) were acquired every 40 min, for 22 h. An oil immersion objective lens at 60× was selected for image capture. Three-dimensional projections [maximum intensity projection (MIP)] of every time point were reconstructed from *z*-stacks using the IMARIS^®^ 8.1 software (Bitplane AG, Zurich, Switzerland). To calculate the parameter, here called *Biovolume* (μm^3^), the MeasurementPro module of the above mentioned software was used. Each image was segmented into two channels, green and blue, analyzed to estimate the biovolume occupied by *Listeria* and *Pseudomonas* cells, respectively. To obtain GFP-*Listeria* cell distribution along the *z*-axis of the *Pseudomonas* biofilm, the Vantage module of IMARIS^®^ 8.1 was used.

### Statistical Analysis

At least three independent experiments were performed and two coverslips were sampled each time (*n* = 6). Data were analyzed using Statgraphics Centurion software (Statistical Graphics Corporation, Rockville, MD, United States). One-way analysis of variance (ANOVA) was carried out to determine whether samples were significantly different at a 95.0% confidence level (*P* < 0.05).

## Results

### Confocal Imaging of the Course of Colonization of *Pseudomonas* Preformed Biofilms by Free Floating L. *monocytogenes* Cells

**FIGURE 2:**
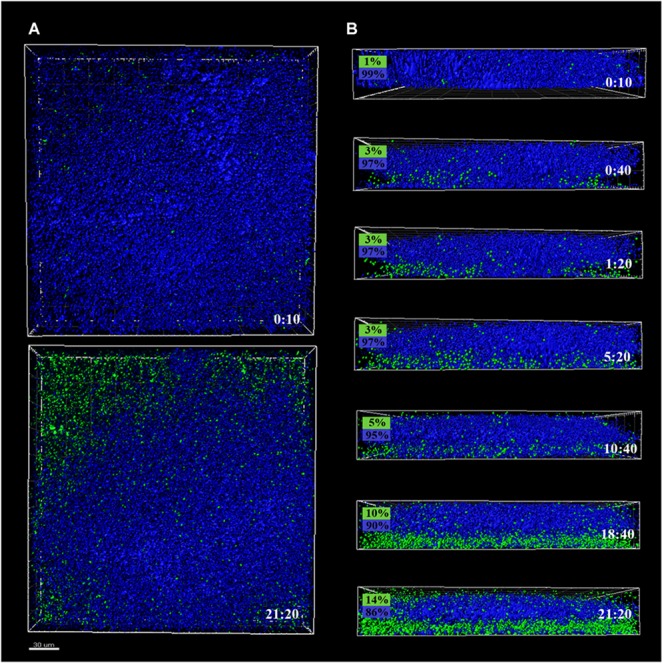
CLSM images of different sections of 48 h *P. fluorescens* biofilms developed at 20°C (*warm*) pre- and after-incubation with *L. monocytogenes* Scott A. *Pseudomonas* cells appear in blue (DAPI) and green cells correspond to the GFP-tagged *L. monocytogenes* Scott A. **(A)** Top image corresponds to the zenital 3D view of *P. fluorescens* biofilm (control) and bottom image corresponds to the same biofilm after 21 h incubation with *Listeria*. **(B)** Snapshots from 2D cross-sections of biofilms (35 μm wide) at several time points along the incubation. In boxes on the left is indicated the percentage of biovolume occupied by each microorganism (*P. fluorescens* in blue and GFP-*L. monocytogenes* Scott A in green). Scale bar = 20 μm.

**FIGURE 3:**
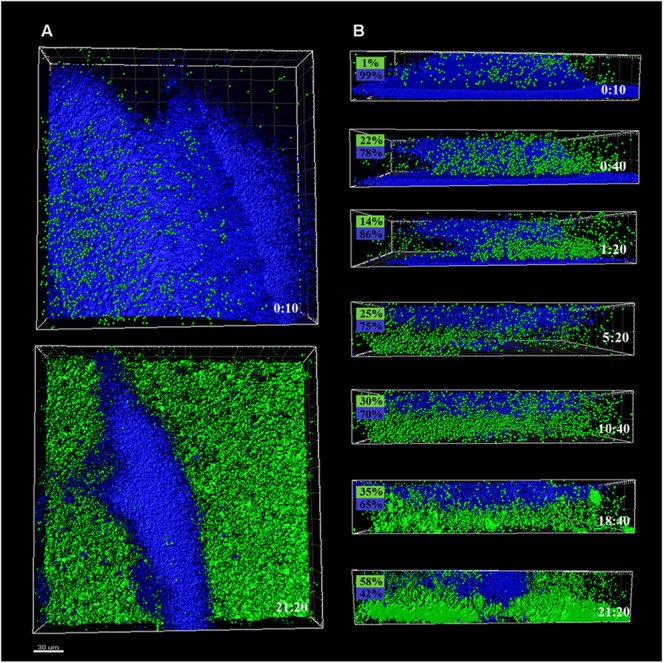
CLSM images of different sections of *P. fluorescens* biofilms developed at 4°C (*cold*) for 10 days, pre- and after-incubation with *L. monocytogenes* Scott A. *Pseudomonas* cells appear in blue (DAPI) and green cells correspond to GFP-tagged *L. monocytogenes* Scott A. **(A)** Top image corresponds to the zenital 3D view of *P. fluorescens* biofilm (control) and bottom image corresponds to the same biofilm after 21 h incubation with *Listeria*. **(B)** Snapshots from 2D cross-sections of biofilms (32 μm wide) at several time points along the incubation. In boxes on the left is indicated the percentage of biovolume occupied by each microorganism (*P. fluorescens* in blue and GFP-*L. monocytogenes* Scott A in green). Scale bar = 20 μm.

Preformed *P. fluorescens* biofilms developed at 20 and 4°C were used as adhesion substrates for GFP-tagged *L. monocytogenes* cells. Interplay of both species was monitored by time-series confocal imaging, considering time zero the moment at which *Listeria*’s suspension was added to the system. *z*-Stacks of these biofilms were captured every 40 min along a 22-h period, to be reconstructed afterward. **Figures 2, 3** display CLSM images of the zenital views (**Figures 2A, 3A**) and snapshots from 2D cross-sections of biofilms at several time points (**Figures 2B, 3B**), using *warm* or *cold Pseudomonas* biofilms for surface conditioning, respectively.

*Pseudomonas fluorescens* biofilms preformed at 20°C occupied more biovolume than those developed at 4°C (2.5 vs. 1.5 × 10^5^ μm^3^, respectively) (**Figures 2A, 3A**). Besides, at 20°C cells had attached more uniformly around the coupon surface. Overall *Pseudomonas warm* biofilms seemed to be more compact than *cold* ones.

Colonization had different outcomes in each case. *Listeria* colonized more efficiently the *cold* biofilms; after 10 h incubation, almost half of the new structure appeared in green, not too different biovolume values being already occupied by green and blue cells (1 vs. 3.5 × 10^5^ μm^3^, respectively). After 24 h, the initial *Pseudomonas* structure was drastically affected by *Listeria*’s presence. *Listeria*, in some way, was able to proportionally displace *Pseudomonas* from the biofilm. Indeed, after 21 h incubation, the biovolume occupied by green cells accounted for 3 × 10^5^ μm^3^.

*Listeria monocytogenes* colonization of *Pseudomonas warm* biofilms was slower. After 10 h of incubation, the biovolume occupied by green cells was negligible compared to that occupied by blue ones (1 × 10^4^ vs. 3 × 10^5^ μm^3^). At the end of the incubation period, the *Pseudomonas* initial structure appeared practically unaltered by the presence of *Listeria*.

**FIGURE 4:**
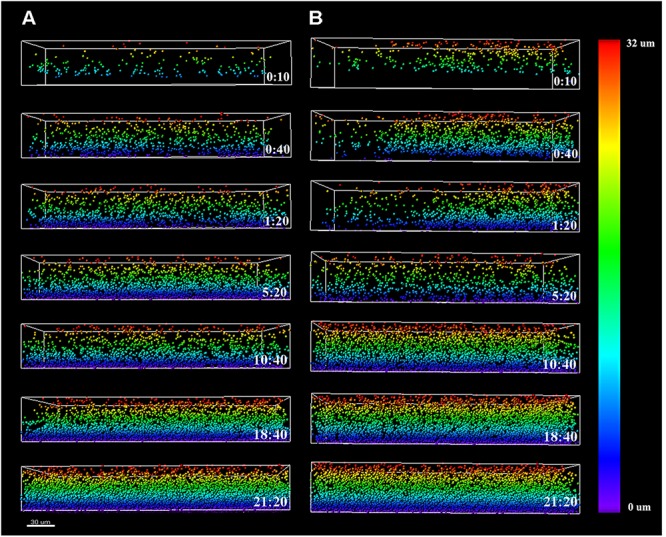
*Listeria monocytogenes* Scott A *z*-distribution throughout *P. fluorescens warm* **(A)** and *cold* **(B)** biofilm structures. Color scale bar on the right stands for *z*-position of *Listeria* cells over time. Scale bar = 30 μm.

To assess the course of colonization, the distribution of *Listeria* cells along the z-axis was examined and quantified using the Vantage module of Imaris (**Figure 4**). The color scale bar shows particle allocation at different biofilm depths. *Listeria* cells were observed to progressively invade the structure previously formed by *P. fluorescens*, either by penetration or by basal infiltration, to eventually occupy preferentially the deepest layers of either *cold* or *warm* preformed *P. fluorescens* biofilms.

### Biofilm Population and Surface Conditioning

**FIGURE 5:**
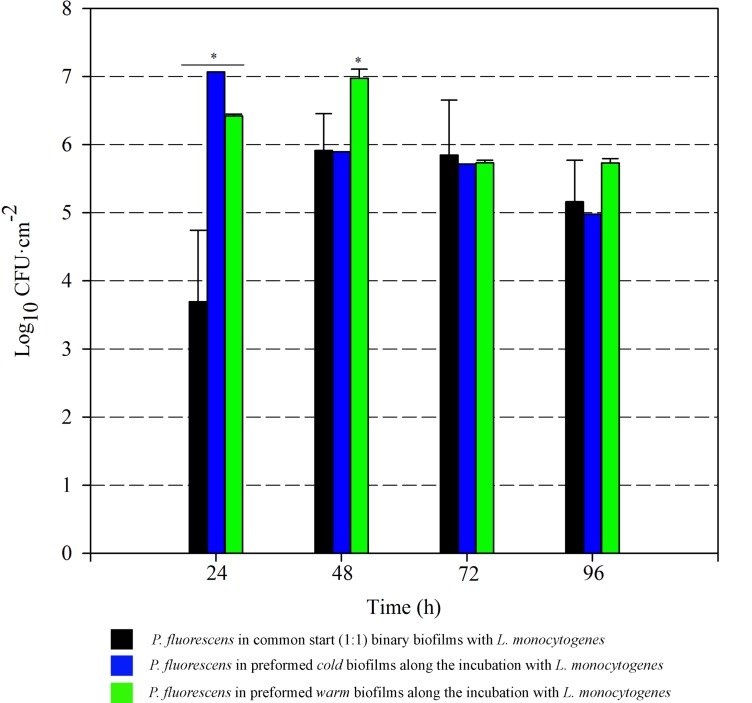
*Pseudomonas fluorescens* attached population over time in different biofilms with *L. monocytogenes*. Asterisks indicate statistically significant differences (*P* < 0.01).

**FIGURE 6:**
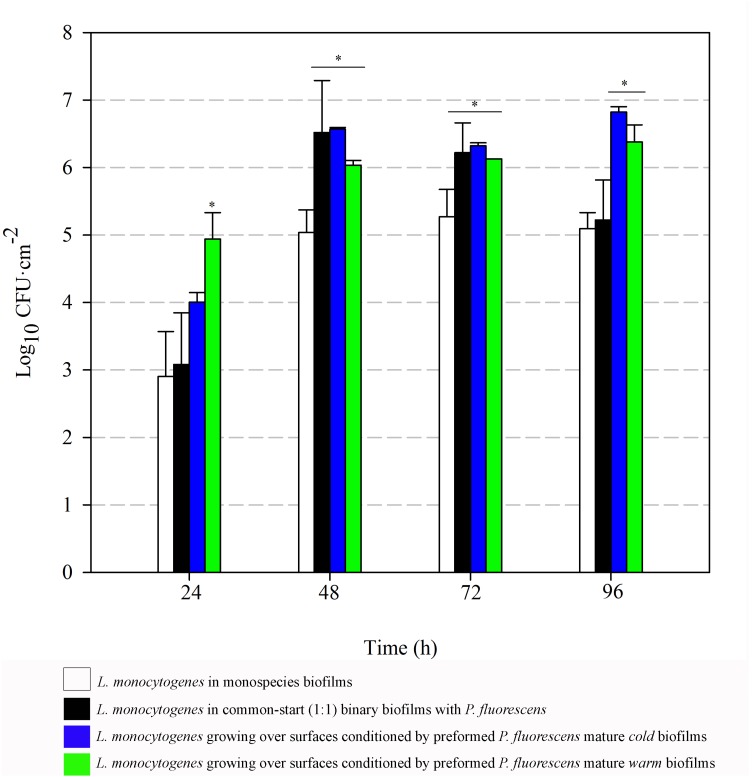
*Listeria monocytogenes* attached population over time in different biofilms. Asterisks indicate statistically significant differences (*P* < 0.01).

To further analyze the advantage effect provided by the already established *Pseudomonas* biofilms on *L. monocytogenes* attachment, the involved populations were quantified. *Cold* and *warm Pseudomonas* biofilms were first developed, and *Listeria* suspension was added for further incubation. In parallel, monospecies *L. monocytogenes* biofilms and *L. monocytogenes–P. fluorescens* common-start binary biofilms (1:1) were developed. Selective plate counts of each species in biofilms are shown in **Figures 5, 6**.

*Listeria* attached population when preformed *P. fluorescens* biofilms were used as a conditioned surface, was on average, 1–2 Log higher than when this organism grew on bare coupons in monoculture (**Figure 6**). A stimulation effect was also observed on the adhesion of *Listeria* when co-cultivated in a proportion 1:1 with *P. fluorescens*, that is, when the two species had a common-start. In that case, however, detachment of *Listeria* cells occurred between 72 and 96 h. When *Pseudomonas* biofilms were conditioning the substrate surfaces, *Listeria* counts were higher at those times, reaching values of more than 6 Log (**Figure 6**). Apparently, *L. monocytogenes* retention was made possible by the presence of preformed biofilms, either *cold* or *warm*.

### Biofilm Biomass and Surface Conditioning

**FIGURE 7:**
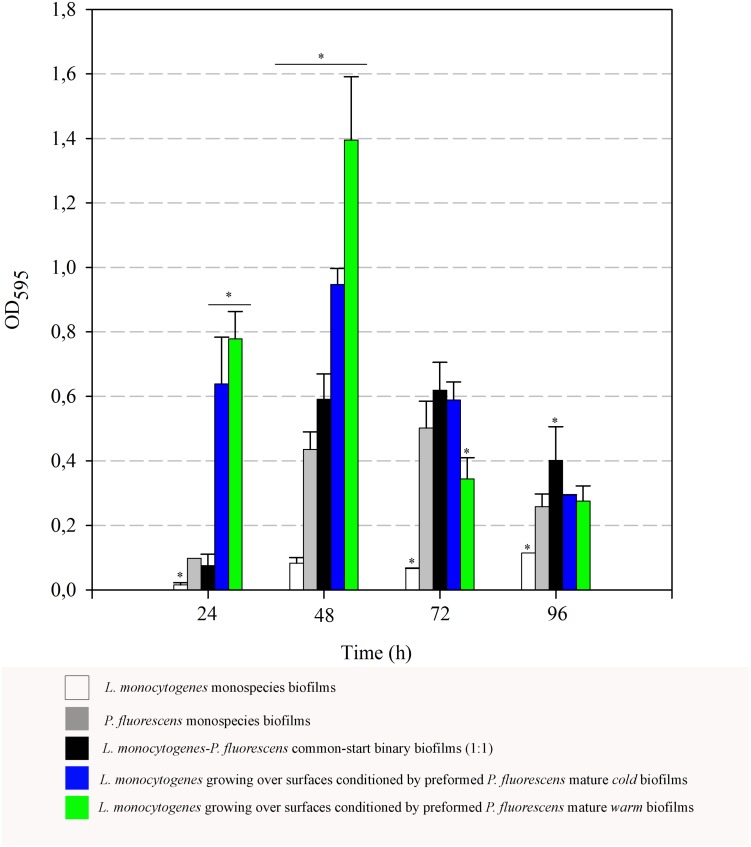
Evolution of biomass (OD_595_) over time. Asterisks indicate statistically significant differences (*P* < 0.01).

In order to complement the results on the retention of *Listeria* here observed, biofilm matrix volume changes derived from the interaction of the two species were also examined. Optical density values (OD_595_) after staining biofilms with Coomassie Blue were thus measured. These OD values integrate cellular and non-cellular components of biofilms, as Coomassie Blue binds non-specifically to protein and carbohydrate. **Figure 7** shows the data corresponding to both monospecies biofilms, binary biofilms from 1:1 cultures and those of *Listeria* growing on preformed *Pseudomonas* biofilms. OD values of monospecies biofilms of *L. monocytogenes* were particularly low, increased rather poorly and reached a maximum value of 0.11 after 96 h. OD values of the common-start binary biofilms were practically similar to the sum of the parts, i.e., contributions of *L. monocytogenes* and *P. fluorescens*, yielding a maximum of 0.62 at 72 h. These data reveal that most of the biofilm matrix volume in this type of consortium is due to the contribution of *Pseudomonas*, whose production seems quantitatively unaffected by *Listeria* under these conditions.

When *L. monocytogenes* was cultured at 20°C on surfaces conditioned by preformed mature *Pseudomonas* biofilms, a strong boost of biomass (cells + matrix) values was observed, particularly at the 48-h samples. At that point, maximal OD values of *Listeria* added to *cold* preformed biofilms were significantly higher than those of common-start binary biofilms (0.9 vs. 0.6). These differences were even larger when *warm* preformed biofilms were used as surface conditioners (1.40 vs. 0.6).

There was a general biomass downshift at late incubation stages. That was larger when preformed *P. fluorescens* biofilms were involved. Minor changes in viable cell count (**Figures 5, 6**) in these *old* biofilms suggest a specific decline in matrix volume.

## Discussion

*Pseudomonas fluorescens* is frequently isolated from food processing plants and is commonly found on the same surfaces with *L. monocytogenes* (Rodríguez-López et al., 2015). Moreover, Langsrud et al. (2016) have recently found that *Pseudomonas* spp. is the dominant genus in the microbiota surviving biofilm sanitation, where the *Listeria* population also represented 0.1–0.01%. The ecological relationships between *Pseudomonas* spp. and *L. monocytogenes* have been studied for many years, both in planktonic environments and in biofilms. Positive, neutral, and negative interactions have been described between these two species, their outcome depending on particular strains and experimental conditions (Breen et al., 1982; Farrag and Marth, 1989; Gram, 1993; Carpentier and Chassaing, 2004; Heir et al., 2018).

The main and more recurrently proposed factor for *Pseudomonas* and *Listeria* cooperation in biofilms has been the ability of *Pseudomonas* to produce large amounts of EPS that would physically engulf *Listeria* cells, or would condition the surfaces as a “first colonizer” before *Listeria* arrives (Sasahara and Zottola, 1993; Hassan et al., 2004). In a previous work, we found that *P. fluorescens* adhered faster than *L. monocytogenes*, although the latter was occupying the deeper layers in mixed biofilms of these species (Puga et al., 2014). This position within the biofilm structure provides an extra protection from physical–chemical damage. Most of the studies involving mixed biofilms use similar levels of microorganisms at the beginning of the incubation, but in real environments, some of them that are free floating might found a pre-established biofilm. Under this scenario, the outcome of the resulting biofilm structure could be rather different. In this work, we wanted to know what happened when planktonic *Listeria* cells found mature *Pseudomonas* biofilms already conditioning a surface.

### *Listeria monocytogenes* Penetration of *Pseudomonas fluorescens* Biofilms

Green fluorescent protein-tagged *L. monocytogenes* planktonic cells did penetrate both types of *Pseudomonas* biofilms, but this penetration was faster in *cold* ones, suggesting planktonic cells found less physical impediment for movement across these cold structures. Structurally, *Pseudomonas warm* biofilms were initially denser than *cold* ones (**Figures 2, 3**). In a previous work, we observed that in dual biofilms, biomass values changed in a temperature-depending manner, being much lower in biofilms developed at 4°C (Puga et al., 2014). As cell numbers were rather similar, this was attributed to the fact that matrix production is affected by cold stress. In this work, OD values for *warm* biofilms were almost double those of *cold* ones at 48 h (0.44 vs. 0.23) (**Figure 7**). As a material, a biofilm exhibits viscoelastic behavior and is considered a gel-like structure, where cells are dispersed into a heterogeneous polymeric matrix (Stewart, 2014). Generally, the gel’s viscosity increases in proportion to the matrix concentration, leading to movement constraints of cells across the biofilm matrix (Stewart, 1998). Matsui et al. (2005) found, using a very different system, that the ability of leukocytes to penetrate the mucus of the lung was restricted in patients with cystic fibrosis whose mucus viscosity is increased by *Pseudomonas aeruginosa* EPS. Houry et al. (2012) demonstrated that several strains of motile bacilli were able to swim across *Staphylococcus aureus* biofilms at a relatively quicker pace. Nevertheless, when *old* biofilms (72 h) were used as substratum, the swimmers’ movement across the biofilm matrix was slower. All the changes in matrix mechanical properties that lead to more viscous and copious matrices are supposed to impede, to a certain extent, the movement of planktonic microorganisms.

Independently of the state of the conditioning *Pseudomonas* biofilm (*warm* or *cold*), we noted that by the end of the cultivation period, *Listeria* cells preferentially occupied the bottom layers (**Figure 4**). The structural pattern in which layers of cells from one microorganism buried those of other species has been previously observed in several consortia (An et al., 2006; Almeida et al., 2011; Habimana et al., 2011; Puga et al., 2014). In co-culture biofilms, this phenomenon, known as blanketing, is generally attributed to the fast rate of growing exhibited by of one of the species in the consortium (An et al., 2006). Nevertheless, while using a pre-established *Pseudomonas* biofilm, this pattern was somehow unexpected, as one might think incoming *Listeria* cells would incorporate at the top layers. Instead, as shown by the particle distribution along the *z*-axis, and the sagittal profile of the CLSM images over time (**Figures 2–4**), *Listeria* cells seemed to drill through *Pseudomonas* biofilms in order to reach the bottom layers of the structure. This “addressed” migration could be due to signaling compounds concentrated in the *Pseudomonas* biofilm matrix. Charlton et al. (2000) found that the biofilm concentration of longer acyl side-chain homoserin lactones (3-oxo-C12 and 3-oxo-C14) was about 4.5 orders of magnitude higher than that measured in open systems (i.e., effluents). Horswill et al. (2007) developed a mathematical model to measure the effect of a hydrodynamic environment on the movement of signaling molecules from the biofilm to the bulk fluid. According to this model, in a closed system, signals produced by a biofilm may induce a quorum sensing response in neighboring bacteria that are not part of such biofilm, whereas in open systems, signals might be continuously washed away. Taking that into consideration, it might be hypothesized that once *Listeria* planktonic cells establish contact with *Pseudomonas* biofilms, they would be somewhat attracted by *Pseudomonas* signaling molecules concentrated at the bottom of the biofilm structure. Nevertheless, we cannot discard the possibility that direct cell contact is also necessary for this observation.

Flagellum-mediated motility is critical for *L. monocytogenes* biofilm formation (Lemon et al., 2007). Previous studies have shown that biosynthesis of flagella in this microorganism is temperature dependent, being motile at 30°C and below (O’Neil and Marquis, 2006). Therefore, flagella expression could have been increased under the experimental conditions used in this work for biofilm formation, acting as the driving force for *L. monocytogenes* to reach the bottom layer in the biofilm matrix. Moreover, *L. monocytogenes* produces certain enzymes, such as chitinases, that might have helped to reach these deep locations inside the biofilm. Chitinases are expressed during the stationary phase so that chitin could be used as a carbon source, although chitin-like structures present in other substrates may serve when the former is not present (Chaudhuri et al., 2010; Paspaliari et al., 2015). The *Pseudomonas* matrix is rich in acetylated polysaccharides, which somehow resemble those of chitin backbone (Kives et al., 2006), a fact that could explain the type of interaction that is taking place between these species within the biofilm.

### Established *Pseudomonas* Biofilms Entrap *Listeria* Cells

*Pseudomonas* stimulated *Listeria* adhesion in both 1:1 co-cultures and cultures with previously attached *Pseudomonas* biofilms. One of the reasons frequently suggested to account for positive effects on the growth of *L. monocytogenes* is *Pseudomonas*’ ability to produce extracellular proteinases that could mobilize essential amino acids, particularly in rich media. Extracellular enzymes, such as proteinases, lipases, and other hydrolases, are very often produced by *Pseudomonas* spp. (Sorhaug and Stepaniak, 1997) and could contribute to nutrient commensalism. Nevertheless, we observed the retention of *Listeria* cells increased if preformed *Pseudomonas* biofilms were already present (**Figure 6**). Moreover, *Listeria* populations remained almost unaltered along the incubation period when coupons were conditioned (**Figure 6**). On the contrary, *Pseudomonas* dispersal was registered (between 1 and 2 Log) after having achieved a threshold cellular density (approximately 7 Log CFU cm^-2^) (**Figure 5**). As the biofilm ages, dispersal of the cells located at the top layers of the structure occurs, while those buried in the biofilm remain practically unaltered (An et al., 2006; Petrova and Sauer, 2016; Puga et al., 2016a). In our case, these top layers were mainly occupied by *Pseudomonas*, as shown CLSM images (**Figures 2, 3**), partly explaining why its cells detach first whereas those of *Listeria* were unaffected by biofilm aging.

Since co-cultivation of *Listeria* and *Pseudomonas* did not attain the same outcomes, it seems that, apart from *Pseudomonas* cells, other components present in the biofilm matrix could be playing an important role in attracting *Listeria* cells to these structures and trapping them more efficiently. In 1:1 co-cultures, both species have to compete at first for nutrients and space. Matrix production is obviously costly, so the process of constructing a solid matrix takes time. Indeed, in 1:1 co-cultures, the highest rate of matrix production took place between 24 and 48 h incubation (**Figure 7**). On the other hand, while incubating *Listeria* with preformed biofilms, this organism found an already stablished biofilm with high cellular density and a copious amount of matrix (*P. fluorescens* 48 h biofilm). This structure was quickly penetrated by *Listeria* as shown in **Figure 4**. In this scenario, the *Pseudomonas* matrix could have become a constraint for *Listeria* movement afterward, explaining why its counts did not change along the incubation period (**Figure 5**). Coufort et al. (2007) found that the basal layer of the biofilm was very cohesive and could resist shear stresses up to 13 Pa. Similarly, Ahimou et al. (2007) demonstrated that cohesive energy increased with biofilm depth. Accordingly, once *Listeria* cells reached these deep layers, most of them will probably become immobilized within the structure, as stiffness also increases with biofilm width (Safari et al., 2015).

Interestingly, the presence of *Pseudomonas* preformed biofilms led to a drastic increase in OD values at 48 h (**Figure 7**). As changes in cell population were negligible, this data suggests that matrix production is somehow over-stimulated by *Listeria*’s arrival to the already established biofilms. Furthermore, this stimulation leads later on to a drastic disaggregation phenomenon, suggesting once the matrix amount reaches a threshold, biofilm dispersal is rapidly induced. This most probably happens by secreting degradative enzymes that are necessary for breaking down polymeric matrices (Li et al., 2014). In our static system, there was no extra nutrient supply, so cellular dispersal phenomena could have been stimulated for this reason. Some authors have reported that, in systems where nutrients are periodically renewed, longer incubation periods are required in order to detect robust biofilms produced by *L. monocytogenes* (Papaioannou et al., 2018; Ripolles-Avila et al., 2018).

In summary, the fast dispersal phenomenon here observed could be somehow frequent in real settings, such as food processing plants. In these environments, biofilm fragments might persist after cleaning and disinfection procedures, and some of them could eventually be colonized by free floating microorganisms. This would in turn led to the formation of the type of biofilms we proposed in this work, in which dispersal mechanisms are really effective. Considering that dispersed cells exhibited a distinct phenotype from both planktonic and biofilm cells (Sauer et al., 2002; Li et al., 2014), the organisms disseminated from these structures would be a persistent source of contamination. Moreover, the capacity of *L. monocytogenes* to penetrate deeper into preformed biofilms could be an added feature to explain its persistence in food processing plants. More studies including persistent strains of *L. monocytogenes* will be necessary to confirm whether the pattern observed here is widespread among this species.

## Author Contributions

CP and BO designed the study, performed experiments and data analysis, interpreted the analyzed results, and prepared the manuscript for submission. ED designed and prepared the GFP-tagged strain of *L. monocytogenes*. CS coordinated research and critically revised the data. All authors listed have made a substantial, valuable, direct, and intellectual contribution to the work, and approved it for publication.

## Conflict of Interest Statement

The authors declare that the research was conducted in the absence of any commercial or financial relationships that could be construed as a potential conflict of interest.
